# Cloud-based email phishing attack using machine and deep learning algorithm

**DOI:** 10.1007/s40747-022-00760-3

**Published:** 2022-06-02

**Authors:** Umer Ahmed Butt, Rashid Amin, Hamza Aldabbas, Senthilkumar Mohan, Bader Alouffi, Ali Ahmadian

**Affiliations:** 1 Department of Computer Science, University of Engineering and Technology, Taxila, Pakistan; 2 Department of Computer Science, University of Chakwal, Chakwal, Pakistan; 3 Prince Abdullah Bin Ghazi Faculty of Information and Communication Technology, Al-Balqa Applied University, Al-Salt, Jordan; 4 School of Information Technology and Engineering, Vellore Institute of Technology, Vellore, Tamilnadu 632014 India; 5 Department of Computer Science, College of Computers and Information Technology, Taif University, P.O. Box 11099, Taif, 21944 Saudi Arabia; 6 Department of Mathematics, Near East University, Nicosia, TRNC, Mersin 10 Turkey

**Keywords:** Phishing detection, Extract feature, Label data, Feature selection, Text processing, Machine learning, Long short term memory (LSTM), Support vector machine (SVM) classification, Phishing dataset

## Abstract

Cloud computing refers to the on-demand availability of personal computer system assets, specifically data storage and processing power, without the client's input. Emails are commonly used to send and receive data for individuals or groups. Financial data, credit reports, and other sensitive data are often sent via the Internet. Phishing is a fraudster's technique used to get sensitive data from users by seeming to come from trusted sources. The sender can persuade you to give secret data by misdirecting in a phished email. The main problem is email phishing attacks while sending and receiving the email. The attacker sends spam data using email and receives your data when you open and read the email. In recent years, it has been a big problem for everyone. This paper uses different legitimate and phishing data sizes, detects new emails, and uses different features and algorithms for classification. A modified dataset is created after measuring the existing approaches. We created a feature extracted comma-separated values (CSV) file and label file, applied the support vector machine (SVM), Naive Bayes (NB), and long short-term memory (LSTM) algorithm. This experimentation considers the recognition of a phished email as a classification issue. According to the comparison and implementation, SVM, NB and LSTM performance is better and more accurate to detect email phishing attacks. The classification of email attacks using SVM, NB, and LSTM classifiers achieve the highest accuracy of 99.62%, 97% and 98%, respectively.

## Introduction

Cloud computing is the on-request availability of personal computer (PC) system assets, particularly information storing and processing power, without an immediate unique association by the customer [1]. The term is mainly used to describe server farms that cover a broad range of customers via the Internet. In cloud computing, there are two public and private clouds. A cloud might be restricted to a single firm (private cloud) or be open to different firms (public cloud). Several attacks are being launched on cloud systems using modern tools and software [2].

Email, another approach to say, “electronic mail” is conceivably the most widely used element of the Internet, close by the web. It permits you to send and receive emails to and from anyone with an email address wherever in the world. The emails use different conventions inside the transport control protocol/Internet protocol (TCP/IP) suite. When designing an email account, you should describe your email address, secret expression, and the server used to send and receive messages. Fortunately, most webmail organizations design your record consequently, so you simply need to enter your email address and the secret code. In any case, if you use an email client like Microsoft Outlook or Apple mail, you might need to configure each record truly. Besides the email address and secret key, you may have to enter the approaching and dynamic mail server and the right port numbers for everybody [3]. Email currently supports hypertext markup language (HTML), allowing messages to be constructed in the same way destinations are. HTML email messages can join, pictures, associations, and cascading style sheet (CSS) designs. You can similarly send archives or “email associations” close-bye messages. Most mail servers license you to send various associations with each message, yet they limit all outsize. Toward the start of the email, associations were usually confined to one megabyte; nonetheless, presently, many mail servers support email associations that are 20 megabytes in size or more.

Phishing is a sort of digital attack that uses a bogus email to steal sensitive client data worldwide, such as accounts login information, MasterCard numbers, and so on. Several spam and phishing email challenges have arisen [4]**.** These measurements have demonstrated that the current enemy of phishing solutions and endeavors is not powerful. Spam might have various structures, including webspam, audit spam, short message administration (SMS) spam, and email spam [5]**.** Webspam tricks web search tools into settling on some unacceptable choices in the positioning of web pages. In survey spam, spammers frequently misuse audits by giving false positive (FP) audits; clients get SMS spam via text messaging; it is tough to annoy clients, but it can also cause financial loss to the specialized organization. Email spam contains an ad or unimportant content, sent by spammers having no relationship with the beneficiary's email. Spam emails are sent from numerous points of view, for example, by utilizing an uncertain worker, utilizing computerized created accounts, newsgroup postings, and utilizing malware to get client addresses. Email spam causes a few threats, for example, digital attacks, fake mail, and loss of lawful messages [6]. For email spam identification, various ML-based methodologies, such as content-based supervised learning, rule-based learning, semi-supervised learning what's more, deep learning, have been proposed.

In the current research, email spam classification uses a substance-based feature set; nonetheless, a set of features is tested, which infrequently satisfies the classification requirements on its own [7]. This paper presents new feature-driven content, client, spam vocabulary, and semantic features [8]. The hacker attacks individual clients, less precise correspondences, loss of work efficiency, abuse of network transfer speed, misuse of document worker extra space as well, computational force, the spread of infections, worms, and trojan ponies, financial losses due to phishing, email phishing, spear phishing, denial of administration (Service) (DOS), index harvesting attack [9]. A spam channel programmer assists the client in labeling an email as spam or ham, indicating whether the email is worth reading or not. It detects and stops spam communications from reaching the inboxes of clients. Based on certain measures spam filter is applied. Although spam does not directly affect client security, it consumes a significant portion of client inbox space [10].

This paper proposes solutions for email phishing attacks using ML algorithms. This attack is used to attack your email account and hack sensitive data easily. Phishing attacks are randomly sent to individuals or groups without planning, but spear attacks target the users. The main role of this attack is to convince the users to read, sign and open this email. After users follow these rules, the attacker attacks his data and hack easily and reads personal data. In this scenario, we used different machines and a deep learning algorithm to classify the result to detect the attack. SVM, NB, and LSTM algorithms are used to detect spear and phishing attacks. Support vector machine (SVM) is an ML algorithm for text classification because of its quick and great implementation. SVM is best to generate execution reports within a nanosecond. Naive Bayes (NB) is an ML algorithm for text classification because of its quick and great implementation. NB is better to generate execution results within a minute. Long short-term memory (LSTM) is a deep learning (D_L_) algorithm well-known text classification because of its rapid and great implementation. LSTM is better to classify execution reports within a minute.

In our work, we apply the SVM, NB and LSTM algorithm to solve the email text phishing attack. This scheme simply extracts the proposed features from the email’s parts (i.e., header, body, text, connections). Their extraction doesn’t need any internet association or the utilization of outer administrations or different systems. We collect spam and not spam email datasets from CSDMC_SPAM online. We train and test the model using email datasets and extract all the.eml (email) datasets to get selected features. After the dataset extraction, we convert it into a CSV file. Natural language processing (NLP) is used to import the CSV and label file to check the spam or not spam dataset and apply text processing to detect the error. We use SVM, NB, and LSTM algorithms to classify the email phishing attacks in the next step. Our model computes the results using different ML and DL algorithms. However, SVM, NB and LSTM show better execution time and system report. The results demonstrate and evaluate the commitment of our features for spam email sites and compare them to existing approaches for phishing and harmful email detection.

The rest of the paper is coordinated as follows: Sect. Introduction explains the brief introduction. Section Literature work discusses the literature review. Section Problem statement explains the security, phishing attack and the problem of the paper. Section Proposed framework discusses the proposed solution, selected algorithm working, classification result, and compare with other and graphs. At long last, Sect. Conclusion and future work discusses the conclusion of the paper.

## Literature work

Phishing attacks are a major threat to individuals and groups in organizations nowadays. Phishing is a method of obtaining a network client's personal information by convincing them to visit a fake website. Back propagation (BP) neural organization is a major heuristic ML technique in phishing site detection systems due to its active learning abilities and superior classifying abilities for certain datasets., backpropagation (BP) neural organization is a significant heuristic ML strategy in phishing site’s location and prevention. However, the inappropriate determination of beginning boundaries, such as the underlying weight and edge, affects the BP neural association into the local least and slow learning confederation. Focusing on these issues, this paper proposes DF. GWO-Back propagation neural network (BPNN) is a convincing phishing site revelation model based on the developed BP neural association and a double component appraisal framework [11]. The two-part evaluation process improves the precision of phishing site identification. This model is differentiated and a couple of existing phishing site revelation models. The test outcomes have shown that our model is precise and strongly adaptable.

The number of phishing attacks has expanded in Latin America, exceeding the operational abilities of network protection experts. The intellectual security challenge proposes using big data, machine learning, and information analysis to improve attack detection reaction times [12]. This paper presents an examination of the investigation of abnormal conduct related to phishing web attacks and how ML strategies are used to remove the issue. This research utilizes data collection and python apparatuses to develop machine learning (ML) for detecting phishing attacks by investigating uniform resource locators (URLs).

Cloud computing is an innovation in information technology (IT) that provides end-users with flexible, virtualized on-demand resources with greater flexibility, lower maintenance, and lower structural costs. These resources are organized by various board associations and distributed via the Internet using well-known frameworks, standards, and strategic plans. The basic advancement and legacy show different flaws and vulnerabilities that might allow attackers to penetrate the system. DDoS attacks are among the best that bring about certified harm and impact cloud execution [13]. This might result in a big loss for cloud structure association data transfer or a significant portion of the server's time [14]. As a result, a DDoS area structure based on the C.4.5 algorithm was constructed to reduce the DDoS threat in this study. We chose various ML algorithms and analyzed the reports to validate our system.

Phishing is a social engineering attack type used to get user-sensitive information such as login information, credit and debit card details, and so forth [15]**.** This paper proposes an original system to effectively perceive phishing sites on the customer side by proposing the best program design. This system uses the rule of extraction construction to eliminate the properties or features using the URL. These cycles are performed out on the client's end, with the help of a modified process plan [14]. Today research has considered ML frameworks to recognize phishing objections, yet they are not in a state to be used by individuals having no specific data. To ensure that these devices are available to every individual, this paper has brought area methods into the program configuration named ‘Embedded Phishing Detection Browser’ (EPDB) strategy to secure the current customer experience while working on the security. We have prototyped this technique to progressively ensure the best security, better precision execution report in the distinctive confirmation of phishing sites.

In this paper [16], the attacker delivers harmful links or attachments through emails that can perform various functions, such as collecting the victim's login credentials or record data. These emails are harmful to receivers, causing financial difficulties and fraud. A combination of defunding and semantic analysis methods was used to detect and prevent the phishing scam. Furthermore, a database of phishing sites is built, and the text, connections, images, and other information on the site are analyzed for design verification. Finally, we evaluated our proposed arrangement and compared it to existing techniques [16]. The results show that our proposed method can effectively manage phishing attacks.

In recent work, phishing is one of the most important risks that a web consumer faces. These attacks continue to cost billions of dollars to organizations associated with the terms, requiring a more effective disclosure technique to reduce the threat [17]**.** This paper proposes a significant use of meticulous imbalance learning in ensembles via selective sampling to differentiate phishing attacks accurately. You can improve your performance using this approach. Furthermore, a comparison report of example machine learning techniques such as random forest (RF), naive Bayes (NB), and decision tree (DT) reveals that the proposed MILES strategy provides higher results and review. These attacks target individuals and relationships' information to arrange deals [18]. Phishing sites provide a variety of indications in their content and web program-based data. The purpose of this research is to run an extremely learning machine-based request for 30 features to the UC Irvine ML repository database that includes phishing site data. In this report analysis, ELM was differentiated from other ML techniques such as SVM and NB and was shown to have the highest raised accuracy of 95.34 percent.

The worldwide spread of the coronavirus disease (COVID-19) pandemic and its significant impact on health, the economy, and almost every aspect of our lives, including how we work, meet, transfer information, communicate, and so on. Furthermore, many new cyberattacks target companies, governments, health care, and other fundamental services [19]. In this paper, attackers try to take advantage of people’s fear of infection, flaws in data collection sensors and Internet of thing (IoT) devices, and effort to explore solutions or assurance. Review the concept of cyberattacks linked to the COVID-19 occurrences. It includes a variety of specialists and social-economic viewpoints and characterizes three layers, end-user, device or sensors, and cloud. End devices are either the source or the destination of data sent over a network. A server is an endpoint device with technology installed that allows it to provide data to other end network devices, such as email and web pages. Sensors are placed at the remote site to identify any changes in the environment and alert the user. After then, the modifications are recorded and uploaded to the cloud server. I combined the proposed model with security threats and protection strategies to counter each layer's network protection threats.

The main goal of the recent study is to develop a new method for detecting phishing attacks and determining how to protect against such threats. The life cycle is how to construct a scrum-based used algorithm for programmed learning, feature determination, and neural networks are shown in this paper. This technique can detect and prevent a phishing attack that has been enlisted within the email worker [20]. For the approval of the obtained results measurement, the blacklist of the fish tank was used as a source of data, which is a dynamic clearinghouse for information and data concerning phishing on the Internet. The direct proof of the idea shows that the actualized highlight determination algorithm removes the qualities of the unessential mail. The neural network algorithm obtains these attributes, resulting in an appropriate level of learning with no redundancies. It also shows the efficiency of the proposed structure.

Previous work discusses different types of attacks and algorithms to solve the problem. Several papers used supervised, unsupervised, and deep learning techniques to solve the attack. Many types of phishing attacks and algorithms are discussed in this paper. This research examined email and text phishing attacks and proposed a supervised and deep learning method to solve them. In this paper, we use the SVM, NB, and LSTM algorithms to solve the problem of email text phishing attacks. Datasets of phish and non-phish emails are collected. To extract features, extract the entire.eml (email) dataset. After extracting the data, convert it to a CSV file. We add CSV and label files in NLP to check the phish or not phish data and apply text processing. We used a machine and the deep learning algorithm to check better results and system reports. Table 1 compares the performance of these approaches.

**Table 1:** Cloud computing using machine learning algorithm literature review

Refs.	Techniques	Year	Advantages	Disadvantages	Objectives	Research
[9]	NN	2018	Reduce phishing issues Improve performance	Insecure data slow system	To secure the sites and personal data to improve the performance using ML strategy	Long term issue
[10]	NN	2019	Website phishing attacks, improve Improve performance Reduce middle attack	Malicious attack Links attack In secure data	To detect URL phishing attacks and apply neural network algorithm to secure data	Still in research
[11]	Decision Tree	2017	DDoS attacks issues using decision tree or C.4.5 algorithm Improve security problem The ML algorithm used to improve performance	Run time error The huge problem in execution	The objective of this paper is to maintain the system daily and check attacks and apply a decision tree to maintain the data	Middle term issue
[12]	Random Forest	2020	Use random forest to detect phishing data Resolve threat and attack issue	Not secure data Phishing attacks Authentication issues	The main role is to secure the client’s login and other data using the algorithm to resolve threat and attack issue	Still in Research
[13]	Gradient boosting	2020	Use gradient boosting to detect malware attack Protect networks	Validation System weakness Data lose Unprotected data	This system has many malware and phishing attack issues and using gradient boosting to detect the attack	Long term issue
[14]	Detection techniques	2020	Malicious to detect website attacks	Session hijacking Security and threats issue	The main point is to detect malicious website security attacks using detection techniques	Middle term issue
[15]	Naive Bayes, Random Forest	2020	AI procedure, Naïve Bayes and random forest to detect malicious and website attack ML used to identify Phishing attacks	Loses personal data In secure sites Data not secure	The most important risk that a web consumer faces and losing personal data and money. NB algorithm is used to identify the attack and resolve it	Long term issue
[16]	SVM, Naive Bayes	2018	Resolve issues using SVM ML strategies to remove attack and threats Improve and secure data	Network not secure Loses personal data	The most important point is consumers face many risks and security issues and lose sensitive data and other detail. This paper applies the SVM algorithm is used to identify and detect the attack	Still in research
[17]	Neural Network	2020	Using data analysis check attacks NN used to resolve attack issues	Cyber attack issue Run time error Malicious security attack	The main goal is to detect personal data and analyze it in the COVID-19 pandemic situation and apply ML algorithms and techniques to resolve the attacks and threats	Still in research
[18]	Neural Network	2019	Detect infected email using NN Improve security and threats problem Secure connection	Phishing Attacks through calls and messages Not Secure network connection	The objective of this paper is to analyze all data and check infected email using NN to secure a connection between sender and receiver	Long term issues

## Problem statement

### Cloud computing

Cloud computing refers to the on-demand use of available computer resources, especially data storage and processing power, without the client handling them directly. Services in large clouds are frequently distributed across multiple sites, each of which is a cloud platform. In cloud computing, there are two public and private Cloud. A cloud might be restricted to a single firm (private Cloud) or be open to different firms (public Cloud).

#### Design of cloud

**Fig. 1:**
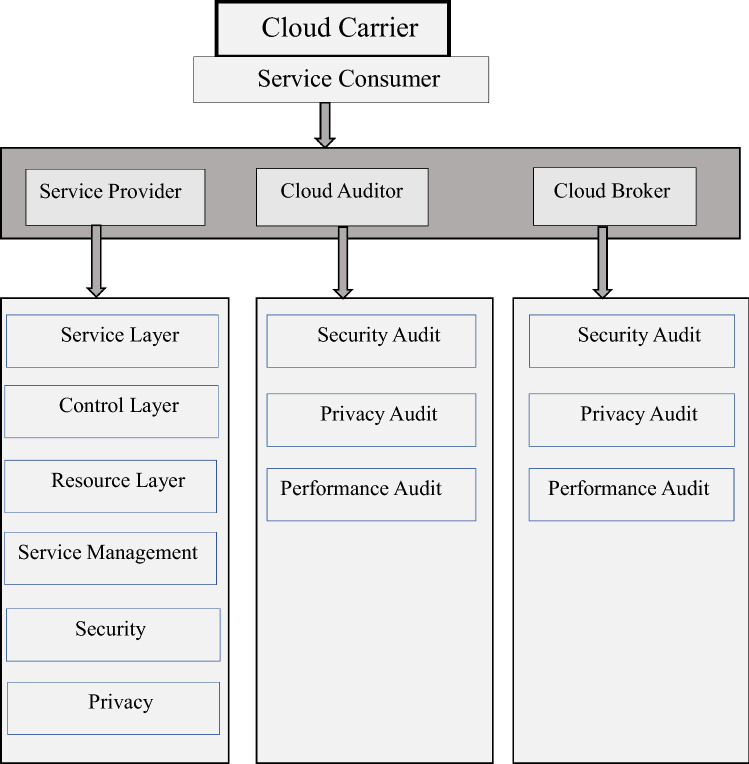
Design of cloud

The cloud computing (CC) strategic plan demonstrates that CC affects the five most important sections and poses a security threat. The CC configuration is shown in Fig. 1, and it comprises a start-to-end reference plan related to the Open Systems Interconnection (OSI) layers. CC is a difficult approach with several weak areas [21]. The following are the components of the CC:

• **Cloud Consumer:** A cloud consumer is a firm that uses cloud-based IT resources provided by cloud providers.
• **Cloud Provider:** A cloud provider is a firm that provides services such as software, network, infrastructure, etc., to cloud consumers.
• **Cloud Auditor:** A public gathering that allows cloud customers to get a free evaluation of their cloud connections, data model activities, execution, and security attacks.
• **Cloud Broker:** The issue that deals with the utilization, execution, and development of cloud services, as well as the development of a link that connects cloud buyers and suppliers.
• **Cloud Carrier:** A service that provides a strategy of cloud relationships from cloud providers to cloud customers.
• **Service Management**: Those service-related functions required to manage and operate cloud services requested by or proposed to cloud users are included in cloud service management. Cloud service management can be characterized from the standpoints of business support, provisioning, configuration, portability, and interoperability requirements.
• **Security:** Cloud security, also known as cloud computing security, is the process of securing cloud-based data, applications, and infrastructure from cyber assaults and threats. It is significant to remember that security is a cross-cutting issue that affects all tiers of the reference model, ranging from physical security to application security. The cloud provider and cloud consumer share responsibilities in general.
• **Privacy:** The technique of securing a company's data in a cloud environment, regardless of where that data is located, whether it's at rest or in motion, and whether it's handled internally or externally by a third party, is described as cloud data protection.
• **Service Layer:** The service layer is the upper layer, where each of the three service models is defined and provisioned by a cloud provider. This is where cloud customers use their cloud interfaces to access cloud services.
• **Control Layer:** The resource description and control layer is the middle layer. This layer provides the system components that a cloud provider utilizes to give and control software abstraction access to physical computing resources. Hypervisors, virtual machines, virtual data storage, and other resource abstraction and management components are often included in this layer to enable efficient, secure, and dependable utilization**.**
• **Resource Layer:** The physical resource layer is the lowest in the architecture, and it contains all of the physical computing resources. Computers (CPU and RAM), networks (routers, firewalls, switches, network cables, and interfaces), storage components (hard drives), and other physical computing infrastructure parts are all included in this tier. It also comprises resources for the physical plant, such as heating, air conditioning (HVAC), and other features.
• **Security Audit:** A security audit is a thorough examination of your company's information system; often, this examination compares the security of your system to a checklist of industry best practices, externally defined standards, or governmental policies. The security auditing process should also involve a check for regulatory and security policy compliance.

• **Privacy Audit:** A privacy audit, also known as a privacy compliance audit, is an evaluation tool that examines an organization's privacy policies and processes in light of current applicable laws and regulatory regulations. A privacy compliance audit might reveal significant liabilities for a corporation.
• **Performance Audit:** A performance audit is an unbiased examination of an organization's operations to see if certain programs or services are meeting their objectives. Because most government entities receive federal funds, performance audits are commonly connected with government agencies at all levels.

### Categorization of cloud threats

Cloud computing is a developing model with incredible opportunities for success and is becoming increasingly well-known; however, it faces various security issues and challenges [5]**.** The classification is done on the Confidentiality, Integrity, and Availability (CIA) Triad and attacks on Cloud Components.

#### CIA cloud security threats

The significant security issues in distributed computing are ordered under confidentiality, integrity, and availability Threats.

##### Confidentiality threats

The objective of 'confidentiality is to ensure data protection by preventing unauthorized disclosure. Only those with real authority to access the data should be allowed access, often called consents on the “need to know” concept. Confidentiality aims to prevent sensitive data from falling into the wrong hands everywhere [22].

##### Integrity threats

This guideline aims to ensure the data’s result, reliability, and validity throughout its life cycle. Data may have value if it is accurate; as a report, appropriate methods should be implemented to prevent the Modification of information, whether extremely slowly or on the move, by unauthorized people or cycles [22]. Whether extremely slowly or on the move, the modification of information by unauthorized people or cycles [22].

##### Availability threats

The term “availability” refers to data being made available to authorized employees as and when they are needed. Monitoring is used to track the performance of the equipment, programming, hardware, and communication channels used to store and process data is critical to maintaining organizational consistency [23].

### Security attacks

**Fig. 2:**
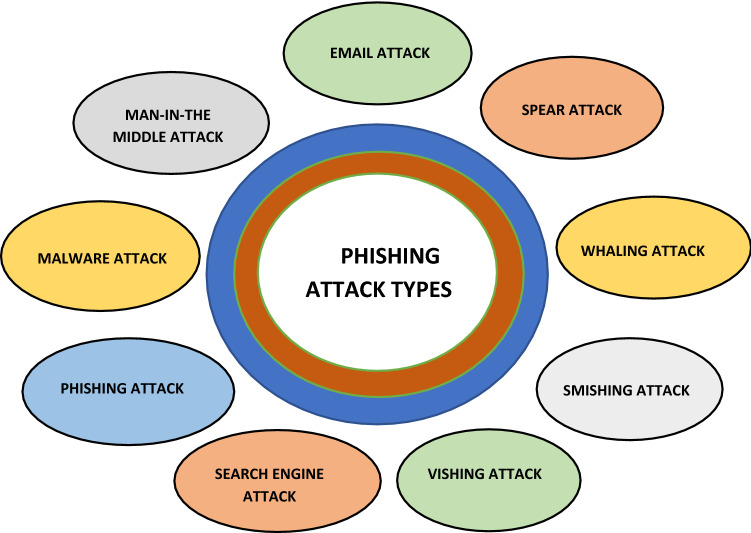
Phishing security attack

This section discusses different types of phishing attacks. Figure 2 explains the phishing attack types.

#### Spear phishing

Spear phishing is a type of phishing attack that targets a specific group of people, such as the organization's structural administrator of a business. You could catch an old boot, a phish, or any other type of phish if you go fishing. If you're doing spear phishing, you’ll choose a specific phish to follow, something along the same lines as the name. The destinations are little more than targets [24].

#### Whaling

Whaling is significantly more focused on the type of phishing than effectively spear phishing because it targets whales, the BIG fish. The CFO, or anybody else in the company, is the target of these attacks. A whaling email may say their company is threatened and needs to tap into the connection for additional information [24]. The association then directs users to a site where they can enter all the important information regarding their relationships, such as their tax Id number and record numbers.

#### Smishing

Smishing is an attack that uses our quality prompting or SMS to make us stand out enough to be noticed. A smash attack is active when a message is received on your phone through SMS that has a relationship to explore or a phone number to call. An SMS that appears to be from your bank and notifies you that your account has been hacked and that you must call or respond right away is an example of a common scenario [24]. By then, the attacker has taken control of your financial balance and demands that you verify your bank account number, social security number (SSN), and other personal information.

#### Fishing

Phishing attacks are similar to phishing attacks, except that the attackers want the customer’s personal information or sensitive company data. As a result, the name has a “v” instead of a “ph.” Many people Microsoft has received a typical attack, and they are concerned about you because you appear to get an infection on your machine. The consumer provides their Mastercard information quickly to increase the type of anti-infection software installed on their laptop. [25]. The attacker now has access to your Mastercard information and has persuaded you to install malware on your computer. From a financial trojan to a bot, the virus could contain anything. The financial trojan will track your online activities to gather more information about you, including your account information and secret password.

#### Email phishing

These phishing emails have most certainly been the most widely recognized type of phishing since the nineties. These are the messages that software developers can send from their email accounts. The email informs the sender that their personal information has been hacked and that they must respond as soon as possible by pressing on the ‘this’ link. It frequently occurs that someone used a translation tool to go through five different varieties and then appeared in English a short time later. Some messages are much more difficult to identify as phishing scams. [24]. It is less likely to be identified as a phishing email when the email is more carefully written in terms of language and sentence structure.

#### Search engine phishing

In any case, web engine phishing, also known as search engine optimization (SEO) harming or SEO trojans, occurs when programmers attempt to rank first in a google or other search engine. It takes them to their (software engineer) site if they are compelling and can persuade someone to click on their link [26]. By then, when you associate with it and enter in sensitive data, they got you. The kinds of sites this could be truly anything; the main competitors are banks, web-based media, shopping, to give some examples.

#### Phishing

Phishing scams are email and SMS messages that focus on the email text, body, etc., as one of the most common social attack types. [27]. Then it tries to manipulate users into sharing personal information, clicking on links to phishing sites, or opening spam email files.

#### Malware attack

A malware attack is when cyber criminals make malicious programming introduced on another person's device without their insight to access individual information or to damage the device, as a rule for financial profit [9].

#### Man-in-the-middle attack

A man-in-the-middle security attack occurs when a responsible party gets involved in communication between a user and an application, either to monitor the situation or to impersonate one of the social gatherings, creating the appearance that regular information exchange is occurring around. An attack aims to steal personal information such as logins, records, and visa numbers [26].

### Email phishing attack problem

Social engineering attacks are carried out in one or more steps and do not necessitate a high level of cybersecurity knowledge. The Social Engineering Life Cycle begins with identifying the victim, collecting information, and selecting attack methods such as phishing emails or phone calls. The second method is known as a Hook, and it involves fooling the victim(s) to get a footing by engineering the target and controlling the contact. For example, in phishing emails, an attacker may get a victim to accept a fake job advancement in the organization by clicking on a malicious or phishing link. The attacker implements the attack and obtains the victim’s information in the third procedure, which is known as a Play, in which the attacker carries out the assault and obtains the victim's data Additionally, the attackers send a cyber-attack when the victim clicks on a malicious link, and it quickly spreads throughout the victim's network. The final phase is called an Exit, which implies that after the Social Engineer has successfully carried out their attack, they conclude the interaction by erasing all traces of malware and covering their tracks so that they do not get detected. Although anti-malware or other software to protect the system, Social Engineering attacks can still occur if the user is not properly trained about the assaults. Baiting, Scareware, Pretexting, Phishing, and Spear Phishing are among the five strategies used.

A phishing email assault is a type of phishing in which attackers send out emails that appear to be authentic and push users to take action. These activities could report the loss of sensitive information, the download of malware, or even financial loss. Phishing is the practice of defrauding people by impersonating a trustworthy entity and taking advantage of human weaknesses to get sensitive information. This could be personal information such as social security numbers or important information that allows the target to gain access to the organization where they work. Phishing can take the form of text messages, phone calls, emails, or even manipulation with search engine results.

Phishing is a social engineering attack frequently used to collect user data, such as login details, emails, personal data, credentials, etc. Phishing and spear attacks are carried out by an individual or a group with the intent to benefit financially. The communications provide data in the form of text, music, video, and other media interactions. Phishing attack's primary objective is the collection of data, which they used by a variety of methods to get data from users easily [6]. The email and web is a rapidly evolving innovation that has connected people worldwide by eliminating all geographical boundaries. However, because of fraudulent and phishing emails and spam messages, users are losing a lot of money and data, and the number of casualties is growing every day.

**Fig. 3:**
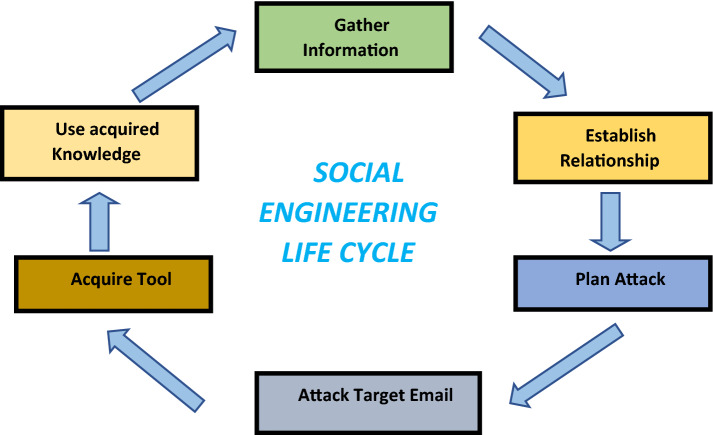
Social engineering life cycle

**Fig. 4:**
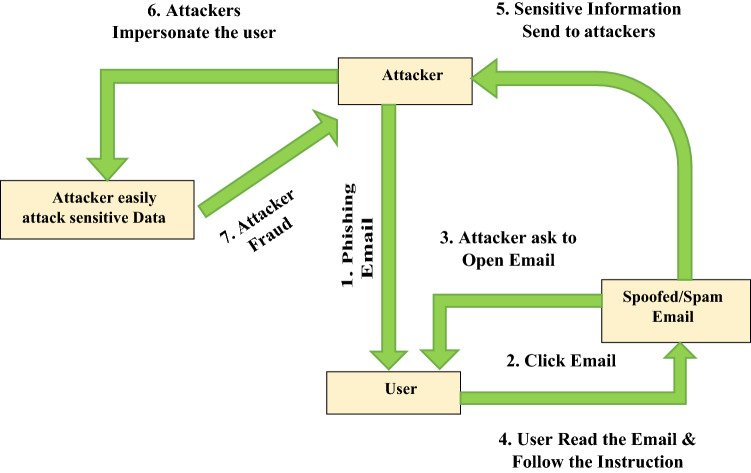
Email phishing attack

The number of phishing emails has increased in recent years due to the number of email clients. Responding to a large number of emails has become more challenging. As a result, numerous researchers have conducted comparison studies to look at different algorithms for classifying shows and their results incorrectly displaying messages using a few measurement systems [28]. Furthermore, for each evaluation of email accuracy and phish or not phish classification, choose an accurate algorithm that provides the best execution time and shows the best result. Figure 3 explains the social engineering life cycle and Fig. 4 discusses the problem of an email phishing attack.

This paper explains email phishing attack issues. Nowadays, it is a major problem all over the world. Everyone does not follow the rules and regulations to read the email. Due to a lack of knowledge, attackers can send phishing emails to users to hack and read your data. In the recent Covid year, all hackers are attacking organizations, institutes, and other email accounts to collect sensitive information. Email phishing attacks have been a major issue in recent years. Attackers can get the data using a different strategy. Every hacker follows the rules to attack emails to collect important data from user sites.

### Effect of phishing attack

**Fig. 5:**
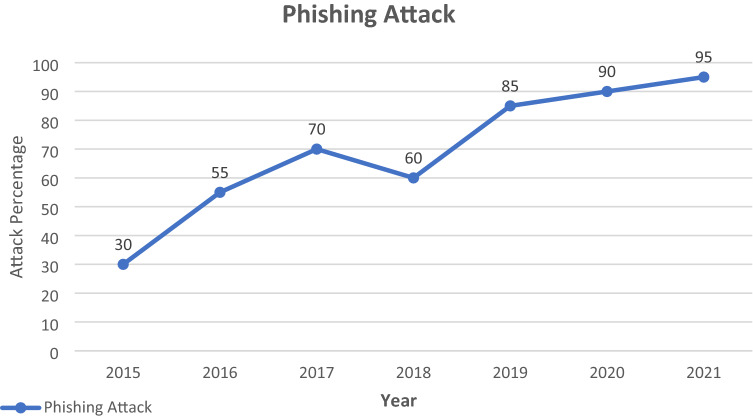
Email phishing attack graph

The diagram below explains the email phishing attacks from the previous and current years. In this paper, use phishing and spear-phishing attacks. Different phishing email attacks in various years are discussed in this graph. In COVID-19 all the world doing work at home. The hacker is active in that pandemic condition, attacking and hacking sensitive data. Because of home-based office work, 99 percent of sensitive data are sent and received via email. Everyone in this circumstance is unaware of the phishing email. So different data hack during this pandemic situation. Figure 5 explain and show the different years of the phishing attack.

## Proposed framework

**Fig. 6:**
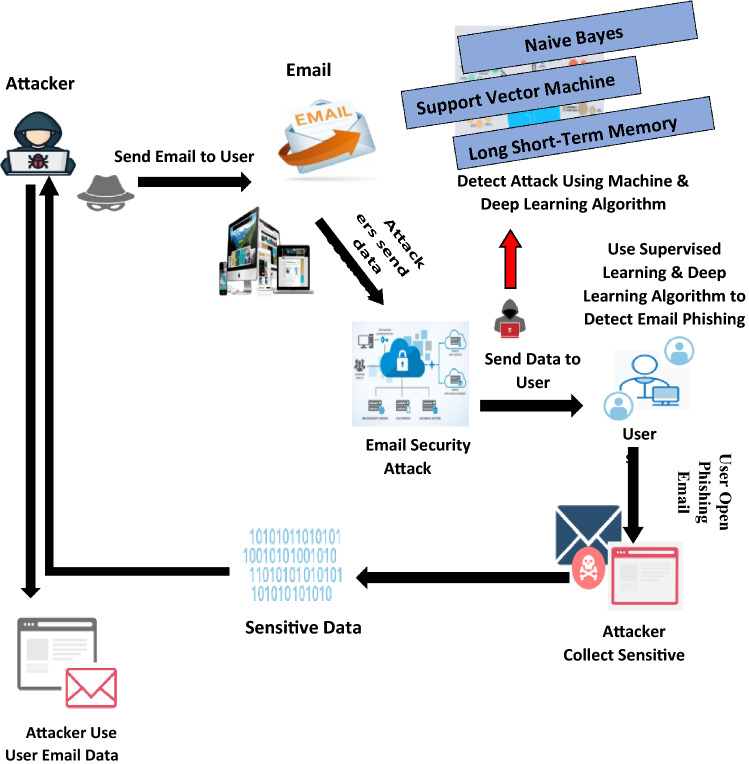
Email phishing attack proposed solution

The proposed solution is based on supervised and deep learning algorithms to detect an email phishing attack. The proposed model consolidates an SVM, LSTM techniques and a bunch of algorithms used to recognize phishing attacks [7]. The proposed approach will research new phishing techniques and include the following phases: data extraction, preprocessing, text processing, train model, text processing, the target variable, split into two trains and test set, classification set, and report using algorithms. Figure 6 shows the overall proposed solution of a phishing attack using machine and deep learning. In the proposed diagram, we have discussed all the procedures step by step. In this diagram, I have shown how the attacker attacks your email and collects your sensitive data. The attacker asks the user to open the email to collect your data and hack your email account. We use this deep and machine learning algorithm to resolve the issue in this scenario. Using LSTM, NB, and SVM to detect email phishing and spear-phishing attacks to control the hacking attack. Using this algorithm show better execution time and generates the report.

### SVM

Supervised learning is a machine learning method in which a limitation is learning to guide a commitment to a yield based on technical data yield sets. One of the essential parts of data science is supervised learning. The supervised ML method is used for construction and unknown sources difficulties [29].

**Fig. 7:**
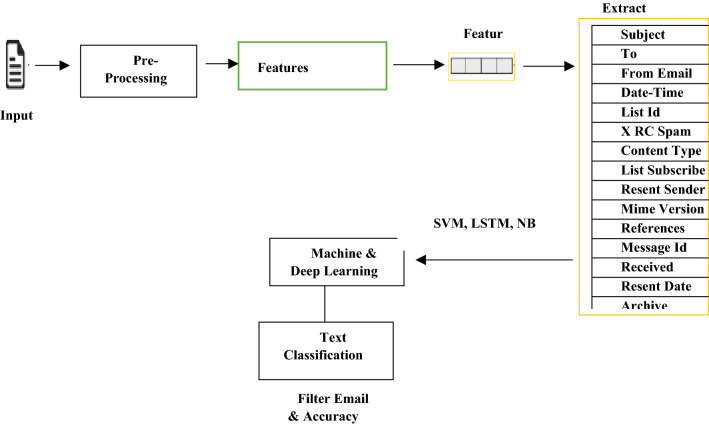
Email text classification using SVM, LSTM, NB

SVM is an ML algorithm for text classification because of its quick and great implementation. As a result of the preparation, it generates a hyperplane, a two-dimensional line that classifies the classes. In a phishing email, various criteria are used to analyze input, such as the existence or absence of a certain phrase and the yield, which is either 1 or 0, and indicates whether the email is spam or not. The SVM approach detects phishing attacks in this research to improve performance, enhance classification reports, and generate results. The general SVM technique is shown in the equation below and the results. Figure 7 shows the procedure of the SVM algorithm. In Eq. 1 linear kernel is the most used kernel for classification. It is used for a large number of features in the dataset. The linear kernel is used for text classification to improve the performance of the dataset. Once the data is linearly separable, when it can be separated using a single line, the Linear Kernel is utilized. It is one of the most often utilized kernels. It is most utilized when a data set contains many features. Text Classification is an example of a feature with a lot of features, as each alphabet is a new feature. As a result, execution measurement, Linear Kernel is commonly used in text classification.

1 $$ {\text{Linear kernel}}: \, K \, \left( {x_{i} , \, x_{j} } \right) \, = \, x_{i} T \, x_{j} $$

#### SVM pseudocode for classification

This pseudocode explains the overall procedure of the proposed solution. SVM (Support Vector Machine) is used to classify the outcome and accuracy result in this pseudocode below. First, extract the data, followed by preprocessing and training the model in this pseudocode. Text processing is used to clean data and split it into 0 and 1 forms, after which the data is trained and tested. The SVM algorithm is used to show classification reports and generate results to identify phishing attacks. Table 2 presents SVM classification results.
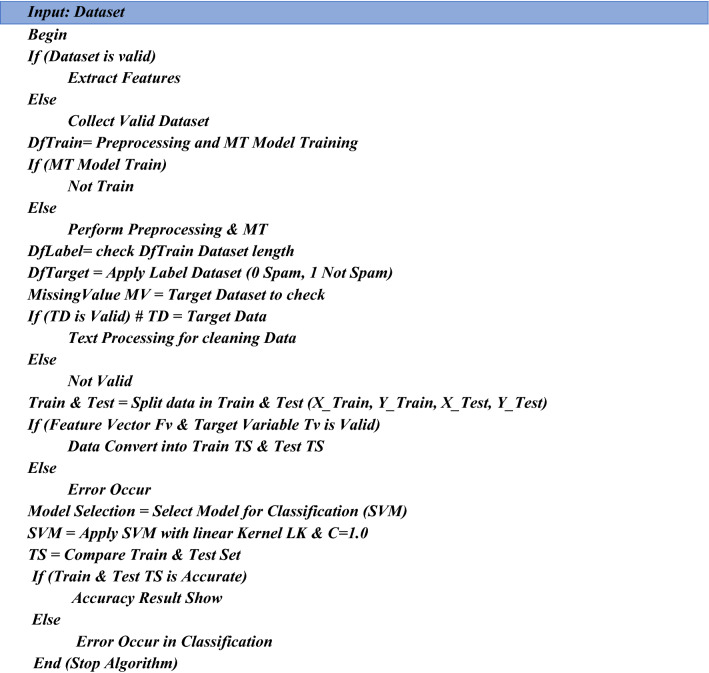

**Table 2:** SVM classification result

Spam/not spam	Precision	Recall	F1-score
0	1.00	0.97	0.99
1	0.99	1.00	0.99
Accuracy			0.99
Macro Avg	0.99	0.99	0.99
Weighted Avg	0.99	0.99	0.99

### LSTM

Deep Learning (DL) is a part of artificial intelligence that simulates the activities of the human cerebrum in processing information and developing designs for use in dynamic settings. Another name for it is deep neural learning or organization. LSTM is a form of artificial recurrent neural network (RNN) in deep learning. It can manage both individual data centers and whole digital data sets [30].

LSTM is a deep learning (*D*_*L*)_ algorithm well-known text classification because of its rapid and great implementation. It produces a hyperplane, a line in two measures that best identifies the classes resulting from the preparation set supplied. In a phishing email, input is handled by several criteria, such as the presence or absence of a certain term, and the yield, which is either 1 or 0, which indicates if the email is a phish or not. The LSTM method is used in this research to identify phishing attacks, enhance performance, and provide better classification-generated reports and results. The general LSTM technique is shown in the equation below and the result. Figure 7 shows the procedure of the LSTM algorithm.

Below Eqs. 2–7 show the difference to classify the report. This paper characterizes the LSTM units at each time step t to be a variety of vectors in *R*^*d*^: an info entryway it, a recall door *f*_*t*_, and yield door ou_*t*_, a memory cell ca_*t*_, and a secret state hea_*t*_. *D* is the quantity of the LSTM units. The filtering vectors *i*_*t*_, *f*_*t*_, and *i*_*t*_ is in [0, 1]. The LSTM progress conditions where cos_*t*_ is the contribution at the current time step, $\sigma$ signifies the strategic sigmoid capacity, and $\odot$ indicates element-wise enhancement. Therefore, the recall door determines how much each memory cell’s unit is deleted, the recall door determines how much each unit is updated. The yielding door controls the opening of the inside memory state. Extra gates, known as the input, forget, and output gates, are used in an LSTM cell to determine whether signals are passed to another node. The recurrent link between the previous and currently hidden layers is denoted by *W*. The inputs to the hidden layer are connected by the weight matrix *U*. Ce is a probably hidden state calculated using the current input and the previous hidden state. *C* is the unit’s internal memory, which is made up of the prior memory multiplied by the forget gate and the freshly calculated hidden state multiplied by the input gate.

2 $$ i_{t} = \, \sigma \, \left( {W_{i} x_{t} + \, U_{i} h_{t - 1} + \, V_{i} c_{t - 1} } \right) $$

3 $$ f_{t} = \, \sigma \, \left( {W_{f} x_{t} + \, U_{f} h_{t - 1} + \, V_{f} c_{t - 1} } \right) $$

4 $$ o_{t} = \, \sigma \, \left( {W_{o} x_{t} + \, U_{o} h_{t - 1} + \, V_{o} c_{t} } \right) $$

5 $$ \tilde{c}_{t} = \, \tanh \, \left( {W_{c} x_{t} + \, U_{c} h_{t - 1} } \right) $$

6 $$ c_{t} = \, f_{t}^{i} \odot \, c_{t - 1} + \, i_{t} \odot \, \tilde{c}_{t} $$

7 $$ h_{t} = \, o_{t} \odot \, \tanh \left( {c_{t} } \right) $$

#### LSTM pseudocode for classification

This pseudocode explains the overall procedure of the proposed solution. LSTM (Long Short Term Memory) is used to classify the outcome and accuracy in this pseudocode below. First, extract the data, followed by preprocessing and training the model in this pseudocode. Text processing is used to clean data and split it into 0 and 1 forms, after which the data is trained and tested. The LSTM algorithm is used to show classification reports and generate results to identify phishing attacks. LSTM classification results are discussed in Table 3.
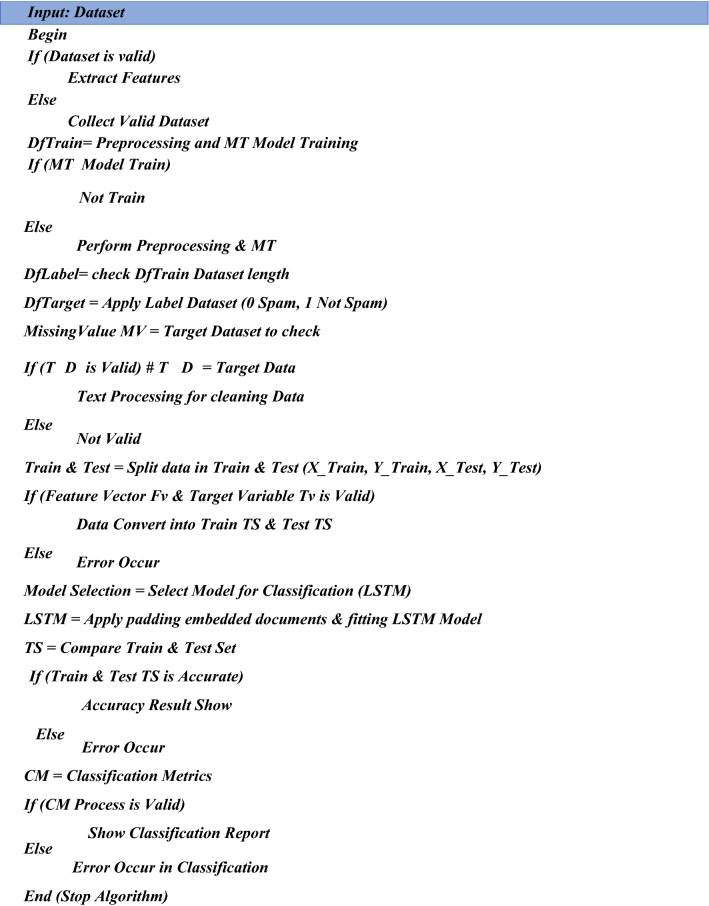

**Table 3:** LSTM classification result

Spam/not spam	Precision	Recall	F1-score
0	0.97	0.96	0.97
1	0.98	0.99	0.98
Accuracy			0.98
Macro Avg	0.98	0.97	0.98
Weighted Avg	0.98	0.98	0.98

### Naive bayes

Supervised learning is a machine learning method in which a limitation is learning to guide a commitment to a yield based on technical data yield sets. One of the essential parts of data science is supervised learning. The supervised ML method is used for both the construction and unknown sources difficulties. Naive Bayes is a fast and simple machine learning technique for predicting a class of datasets. It's suitable for both binary and multi-class classifications. In comparison to other execution measurements, it performs well in multi-class estimations. It is the most often used method for text categorization. Figure 7 shows the procedure of the NB algorithm.

#### Naive bayes pseudocode for classification

This pseudocode explains the overall procedure of the proposed solution. NB (Naïve Bayes) is used to classify the outcome and accuracy in this pseudocode below. First, extract the data, followed by preprocessing and training the model in this pseudocode. Text processing is used to clean data and split it into 0 and 1 forms, after which the data is trained and tested. To identify phishing attacks, the Naïve Bayes algorithm is used to show classification reports and generate results as shown in Table 4.
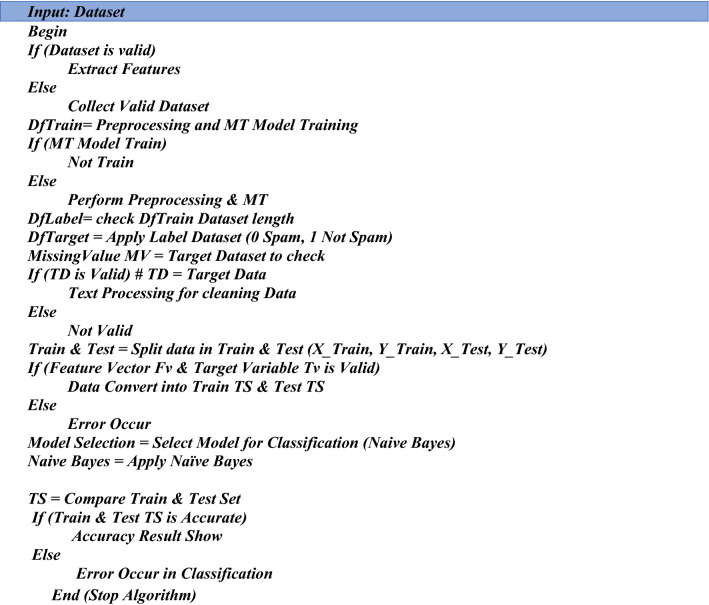

**Table 4:** Naive bayes classification result

Spam/not spam	Recall	Precision	F1-score
0	0.97	0.95	0.97
1	0.97	0.96	0.97
Accuracy			0.98
Macro Avg	0.97	0.96	0.97
Weighted Avg	0.97	0.97	0.97

#### Proposed algorithm flowchart

**Fig. 8:**
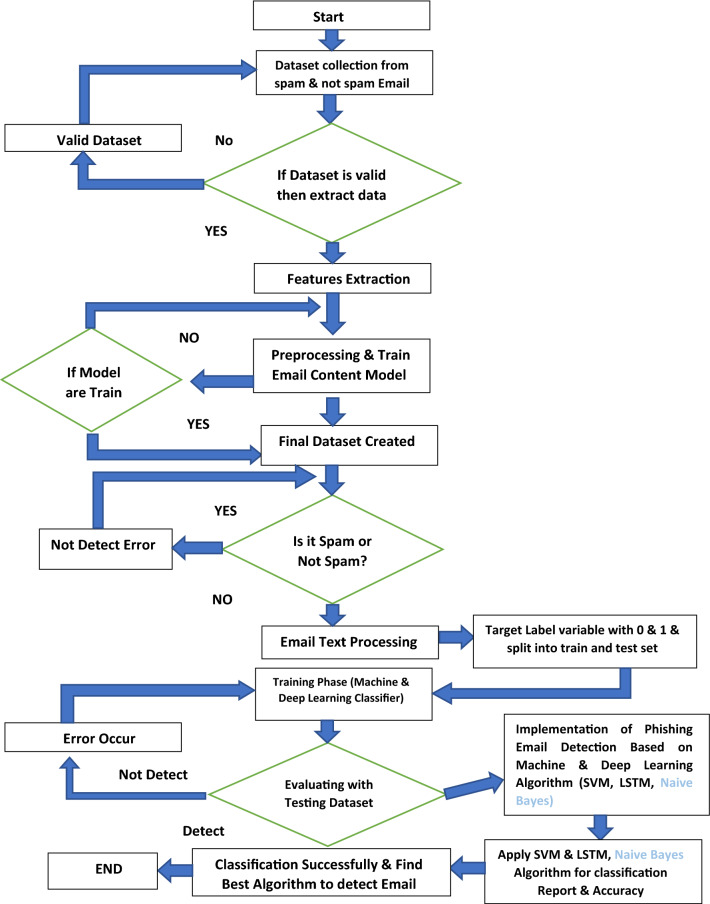
Email phishing attack classification proposed solution

This flowchart discusses the overall working of the email phishing attack classification using machine and deep learning algorithms. We have discussed the LSTM, NB, and SVM algorithm step by step to solve the phishing attack problem. The phishing email classification technique is shown in Fig. 8 to detect an attack. The diagram below shows the whole process of our work.

### Result and discussion

#### Dataset preparation

This section, discuss feature selection and extract of the dataset. Different types of features are selected from the dataset and extracted using the code in this paper. To execute and prepare the dataset, use the output command. The first step is to email (.eml) dataset files, save them in any folder, and rename data. This folder contains a variety of data. The dataset is tested and trained and generate results in the output folder. Choose a different variety of features. You choose the features first, then type in the code and select the dataset source and destination. Run the code using the extract. Pay file to extract selected features after adding all libraries. Run the code to ensure that the source and destination are working properly, and then save the CSV file to the appropriate folder. So, convert all. Email files to CSV files easily. This section collects the dataset online and adds some more (.eml) email and modifies it. After combining the dataset, we check all the datasets deeply and set the label file. There is a four thousand plus email (.eml) dataset and we select some features from this dataset and extract them.

#### Preprocessing phase

There are two steps here at the pre-preparing stage. The first stage is to identify which elements of each email's text and header should be deleted; these components reflect the email’s numerous attributes. To speed up the layout and modification of the classification model, the following phase involves selecting the best solutions from the set separated in the previous step. Data preprocessing is a phase in the data mining and data analysis phase that turns raw data into a format that computers and machine learning can understand and evaluate. Cleaning, instance selection, normalization, transformation, feature extraction and selection, and so on are examples of data preprocessing. The final training set is the system report of data preparation.

- Data cleaning/cleansing
  Data in the real world is frequently incomplete, noisy, and inconsistent. Data cleaning methods aim to fill in missing values, smooth out noise while identifying outliers, and fix data discrepancies.
- Data integration
  As in data storage, data integration is involved in data analytic tasks that combine data from numerous sources into a consistent data repository. Multiple databases, data cubes, and flat files are examples of these sources. Schema integration is an important consideration in Data Integration. It's a hard situation.
- Data transformation
  Data is translated into mining formats that are relevant to the situation. The following steps are involved in data transformation:
- In Normalization, the attribute data is scaled to fall inside a limited predetermined range, such as − 1.0 to 1.0 or 0 to 1.0.
- Smoothing is a technique for removing noise from data. Clustering, grouping, and regression are examples of such procedures.
- Aggregate is the process of applying summary or aggregation procedures on data. Daily sales data, for example, might be combined to calculate monthly and yearly totals. This phase is commonly employed while building a data cube for data analysis at several granularities.
- Using concept hierarchies, low-level or primitive/raw data is replaced with higher level ideas in generalizing the data. Categorical qualities, for example, are generalized to higher level notions such as street, city, and nation. Similarly, numeric attribute values can be mapped to higher level notions such as age, classified as youthful, middle-aged, or senior.
- Data reduction
  Complex data analysis and mining on large datasets might take a long time, considering such a study unaffordable or impossible. Data reduction techniques are useful for analyzing a reduced representation of a data collection without compromising the original data's integrity while still yielding qualitative knowledge.

**Fig. 9:**
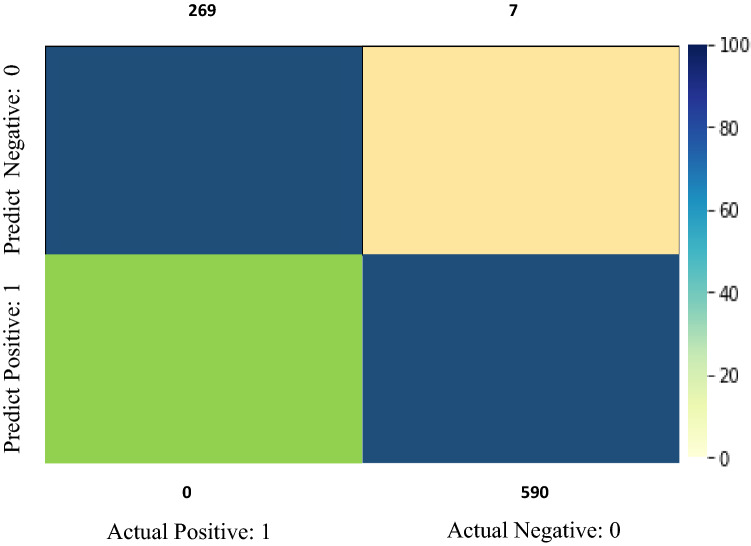
SVM confusion matrix

#### Text processing

It is a critical stage in any text analysis application. There will be numerous unhelpful substances in the news that can restrict the maintenance of an ML and DL model. The machine and deep acquiring model would not work properly unless removed. Use the dataset and text processing for classification at this stage and generate the result. The text processing procedure gets effective when the algorithm is being used to classify reported.

#### Experimental result

This paper discusses tree-based, SVM, NB, and LSTM classification algorithms. Different execution measures are used to evaluate the performance of different classifiers, as shown in this section. SVM, NB, and LSTM classify the dataset with the highest precision of 99.62 percent, 97 percent, and 98 percent, respectively. Figure 9 shows the SVM confusion matrix result. Equation 8 calculates the result measurement using the accuracy formula. The following exhibition measurements are utilized for assessing our model:

8 $$\mathrm{Accuracy }=\frac{TP+TN}{TP+TN+FP+FN}$$

- **Precision**: This is defined by the minimal number of recoverable elements that are beneficial. In our situation, the number of messages that are effectively delegated are phished. The precision result of this approach is shown in Eq. 9.
  9 $$\mathrm{Precision }=\frac{TP}{TP+FP}$$
- **Recall:** It is defined as the percentage of phishing messages that are phished from the dataset in terms of the number of relevant objects recovered compared to the hundreds of significant items in the dataset, i.e., the percent of phishing messages that are collected are phished from the dataset. Recall Eq. 10 was used to validate the outcome after the precision report.
  10 $$\mathrm{Recall}=\frac{TP}{TP+FN}$$
- **F-measure**: It is characterized as the consonant mean of accuracy and review. In below Eq. 11 used the precision and recall formula to find an F—measure.
  11 $$\mathrm{F}-\mathrm{measure}=\frac{2\times Precision\times Recall}{Precision+Recall}$$

- **True Positives (TP)**—True Positives (TP) happens when anticipating a perception has a place with a specific class and the perception has a place in that class.
- **True Negatives (TN)**—True Negatives (TN) happens when anticipating a perception doesn't have a place with a specific class and the perception doesn't have a place in that class.
- **False Positives (FP)**—False Positives (FP) happens when perception has a place with a specific class; however, the perception doesn't have a place in that class.
- **False Negatives (FN)**—False Negatives (FN) happen when predicted perception doesn't have a place with a specific class; however, the perception has a place in that class.

**Fig. 10:**
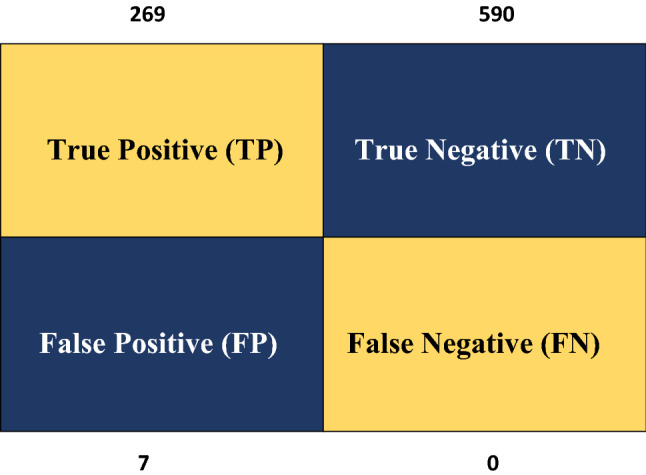
Confusion matrix TP, TN, FP, FN

The confusion matrix diagram shown in Fig. 10 shows the outcome in several sections. The TP, TN, FP, and FN rates are shown in this diagram. You can use this matrix to divide the data into multiple forms and then use code to determine whether the data is TF, TN, FP, and FN. To discuss the various data rates, see the confusion matrix above.

### Selected algorithm for classification

**Fig. 11:**
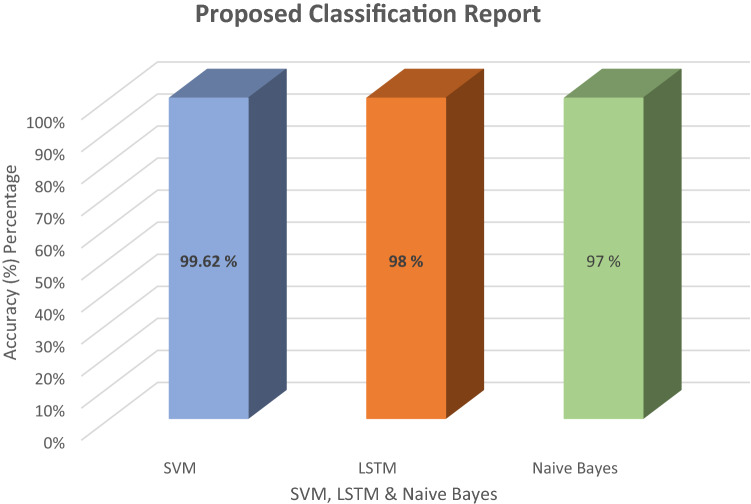
Classification report

**Fig. 12:**
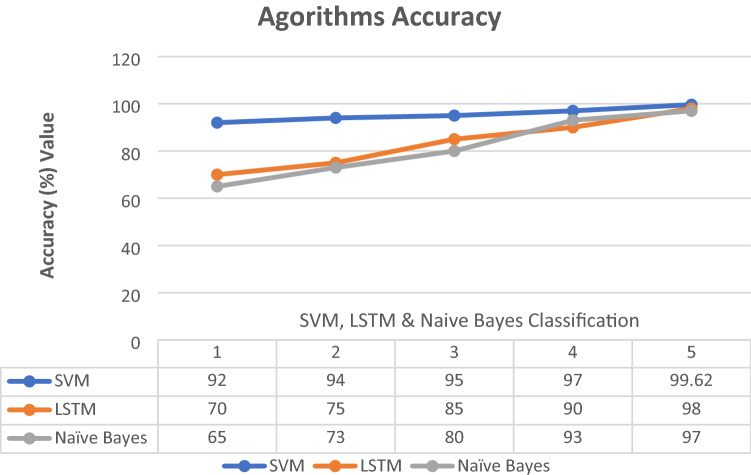
Algorithm accuracy result

In this diagram, Fig. 11 shows the classification between these algorithms SVM, NB and LSTM. To classify the dataset, the approach below utilizes several phases. This paper describes the various algorithms that may be used to experiment with the dataset. Utilizing machine and deep learning techniques, a classification report was generated. The report of SVM, NB, and LSTM is shown in this figure. LSTM has a 98 percent accuracy after classification, NB has a 97 percent result after classification whereas SVM has a 99.62 accuracy. This algorithm detects the attack with the highest level of result. The accuracy of these selected machines and deep learning methods is shown in Fig. 12. This figure discusses the classification report. This paper explains how to use the support vector machine and long short-term memory algorithm to determine the outcome when a dataset is provided. These proposed lines in the figure, describe the system report after phishing and spear attack detection. Evaluate the graph's performance and concluded that this research has the best and properly checked phishing data.

### Evaluation and performance of algorithm

**Fig. 13:**
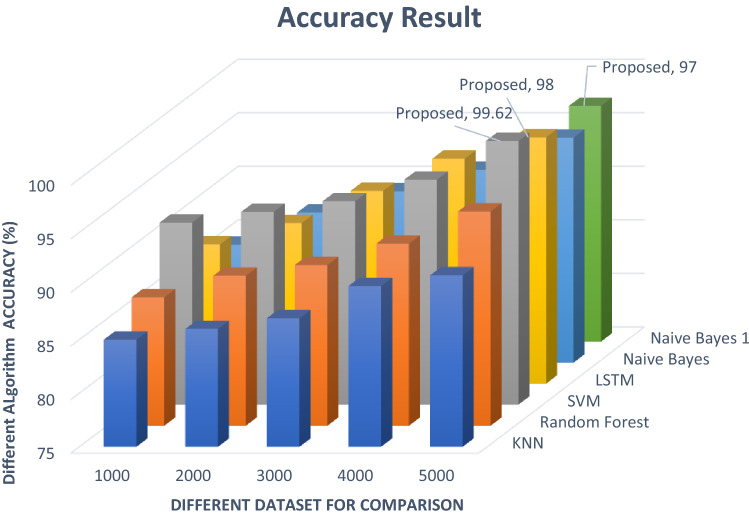
Different algorithm classification

**Fig. 14:**
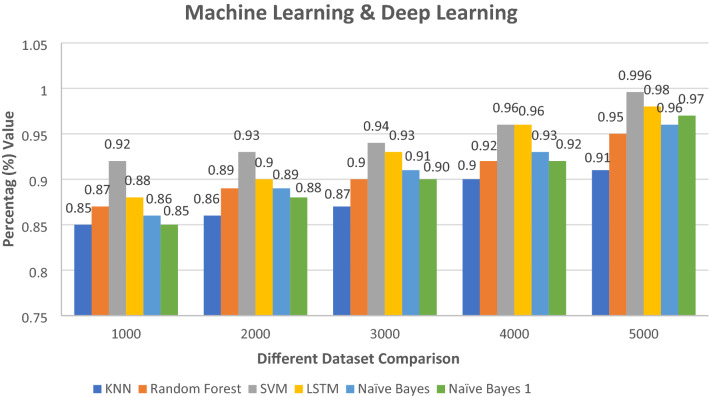
Different algorithm classification result

Figure 13 analyzes all the algorithm results and compares them with our report. These figures demonstrate the classification and accuracy report in comparison to other datasets of various sizes. Analyze and filter the data throughout all datasets. To begin, determine which dataset is the most efficient. First, utilize code to extract email data E_d_, then check that all extract features are strong and appropriate for classification. The graphs below show that SVM, NB and LSTM are greater in classification and generate the result. The SVM algorithm classification result is 0.996 in nanosecond NS and the NB, LSTM algorithm classification generates the report is 0.97 and 0.98 min M. Figures 13 and 14 demonstrate our execution time, report, and compared to the results of other algorithms. Different types of datasets are shown in this diagram for comparing our algorithm system result with other execution time reports.

### Comparative analysis

In today's world, email is important for communication; web users are connected, and email is necessary for online communication. The phishing email is a major issue for authorities to analyze and eliminate. Phish communications are received in large numbers, and they include trojans, contaminations, and malware are used to execute phishing attacks [31]. This paper used several depiction approaches to perceive phish mail and delete it, claiming that moving toward messages is spam mail or ham. In backward probability, naive Bayes classifiers are used, and choice tree methods such as random, reduced error pruning (REP), random forest, and J48 decision tree classifiers are used. The evaluation and the implementation of the system report are done using weak writing computer programs. At the collecting time of Weka programming, game plan algorithms are utilized to find phish emails in Weka Tool. These papers are important in eliminating viruses, trojans, malware, and other dangers, such as phishing threats and fake endeavors in the mails. Therefore, Random Tree produces the best execution time for phish mail classification.

Email is one of the most popular ways to communicate on the Internet. Because of the rapid growth of the Internet, the utilization of email correspondence for business, personal, and other purposes has entered an era of electronic information in increasing demand due to the fast development of internet technology. As a generated report, the data must be prepared for machine learning strategies to be executed more effectively. In machine learning applications like classification, clustering, and expectation, the preprocessing stage is recommended to reduce the amount of data. [32]. In the field of the email class, this paper offers a new data preparation strategy for imbalanced data to evaluate the effects of different preprocessing strategies on various ML classifiers. According to the accuracy analysis, the proposed technique is based on the precision of all the ML classifiers used in this study. According to the system report of this study, the proposed technique achieved 90.39 percent exactness in the achievement rate of logistic regression.

In the COVID-19 pandemic situation, individuals are implemented to take on the 'home' strategy. These days, the Internet has become a strong tool for establishing social bonds. The increasing dependence of people groups on advanced technology provides opportunities for fraud. Phishing is a cybercrime that involves stealing clients' credentials from online platforms such as web-based banking, online business, online homeroom, digitalized commercial centers, etc. Phishers create fake pages that look almost exactly like the original and send spam messages to attract clients. Once an online customer views the fake pages, phishing keeps records of their credentials. Researchers have offered effective tools like boycotts, whitelists, and antivirus software to identify phishing pages. Attackers are always coming up with new ways to get past digital protections by exploiting human and organizational flaws. This study uses a deep learning approach to provide an information-driven system for detecting phishing internet pages. A multi-facet perceptron is used, also known as a neural network (NN) [28]**.**

Cybercriminals have used phishing emails to successfully attack several significant data systems in recent years, resulting in huge damages. The detection of phish mail from large amounts of data has been brought to the public's attention. In this paper, phishing mail is becoming increasing day-by-day, and the current identification techniques can’t correctly check for phishing email attacks. This paper proposed an LSTM based phishing recognition strategy for many emails information. The new strategy incorporates two major phases, the test extension stage, and the testing stage under suitable examples. In the example extension phase, consolidate k-nearest neighbors (KNN) with *K*-Means to extend the informational preparation, collection, allowing the size of preparing tests to address top-to-bottom learning issues. In the testing phase, first preprocess these examples, counting generalization, word division, and word vector age. Then, at that point, the preprocessed information is utilized to prepare an LSTM model. After that, the preprocessed data is used to build an LSTM model. Finally, organize the spam messages using the prepared model. This paper investigates the performance of the suggested approach and classification reports, concluding that our phishing attack detection system may achieve an accuracy of 95%.

**Fig. 15:**
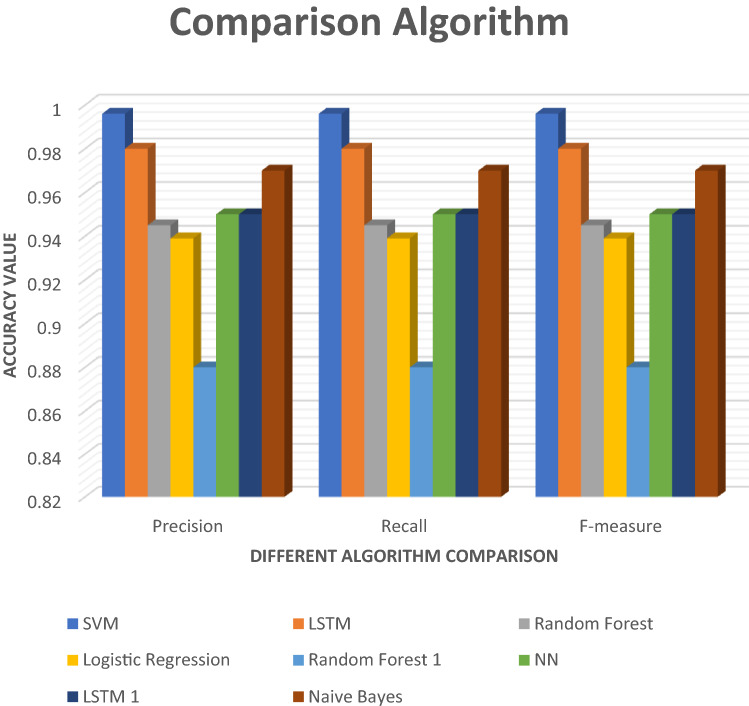
Different algorithm comparison

Figure 15 shows all the Precision, Recall and F-measure execution reports in this paper. To classify email phishing attacks, this paper use machine learning, and deep learning algorithms. Two different approaches are utilized in this research to detect the attack. SVM, NB, and LSTM algorithms are used to detect email attacks and provide classification reports with high results. Compare our execution results to those of other research to see how precise, recall, and *F*-measure are. Our experimental report shows better research in terms of accuracy and classification performance. In this study, we apply our chosen algorithm to resolve email phishing attacks efficiently (Table 5).

**Table 5:** Comparison of precision, recall, *F*-measure

Classifier	Precision	Recall	F-measure
SVM	0.996	0.996	0.996
LSTM	0.980	0.980	0.980
Random forest	0.945	0945	0.945
Logistic regression	0.939	0939	0.939
Random forest	0.88	0.88	0.88
NN	0.95	0.95	0.95
LSTM	0.95	0.95	0.95
Naive Bayes	0.97	0.97	0.97

**Fig. 16:**
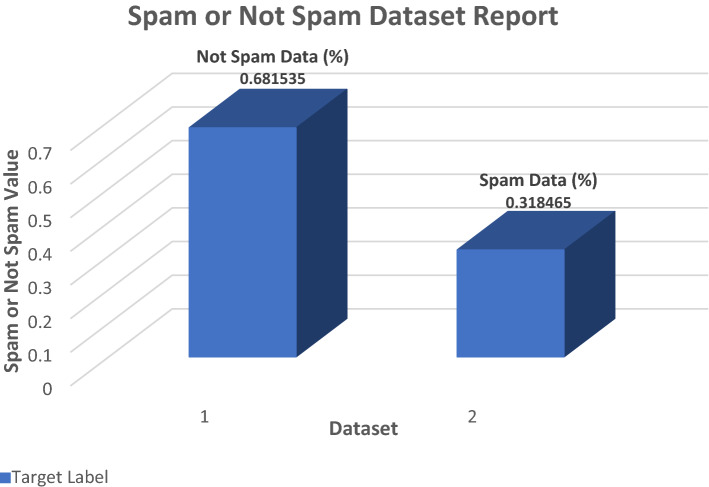
Label dataset spam or not spam

**Fig. 17:**
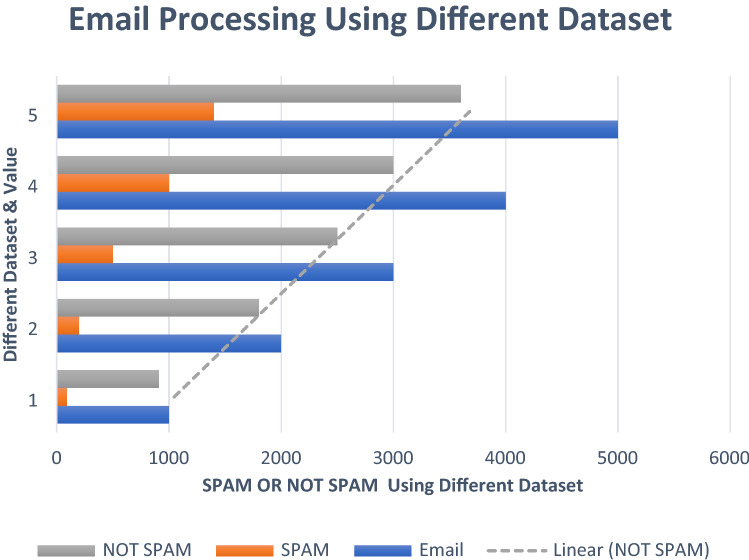
Spam or not spam

**Fig. 18:**
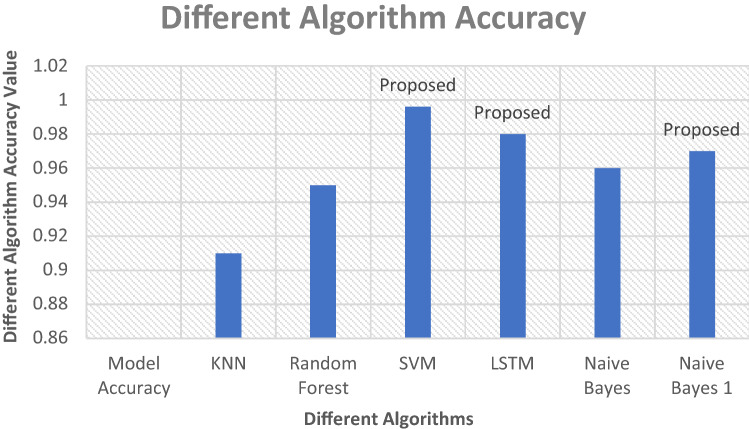
Train model gain

**Fig. 19:**
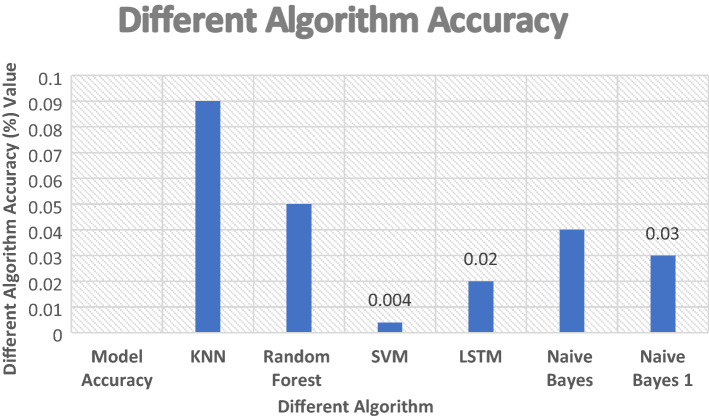
Train model loss

**Fig. 20:**
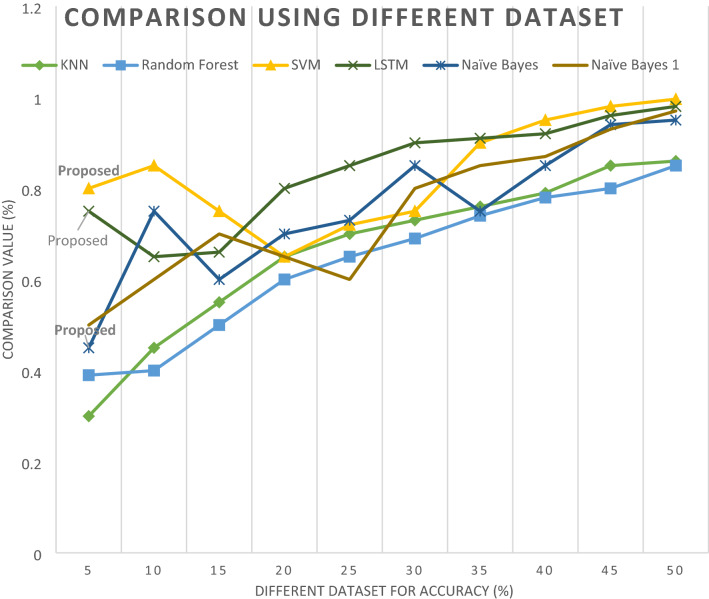
Accuracy using different dataset and algorithm

### Proposed spam or not spam data evaluation

The extracted feature dataset is shown in this figure. The diagram below shows how many email datasets are spam or not. Figures 16 and 17 illustrate the accuracy of the email dataset. To begin, gather information from a different area. Also, save spam and non-spam email datasets to a folder when collected. Choose a variety of email features to include in the code. After adding unique features to code, use the extracted command to extract the selected features S_f_ and store them in a CSV file. CSV may be easily executed and saved in the destination folder if the source and destination are appropriate. I utilized the train label *T*_*L*_ file to identify whether email data was spam or not. Once you've applied the code and added the features you want, you'll have to execute it. After running the code, the output displays if the email is spam or not. As an exhibition measurement, 69 percent of the data in this dataset are not spamming, whereas 31 percent are spam. The figure below shows the percentages of spam and non-spam emails.

#### Evaluation of train model gain and loss

The train and test data (T_D)_ are discussed in this figure. The model's gain and loss are shown in this graph. You should choose phishing and non-phishing email datasets before evaluating them. After extracting the dataset E_d_ adds label data file le_d_ in the code. To identify and assess the algorithm's performance P, this paper utilizes two different methods. This paper does the classification using machine learning SVM, NB, and deep learning LSTM algorithms. Train model T_M_ is utilized to assess the classification result given in these figures. As a result, Figd. 18 and 19 show the train model dataset does gain and loss classification generates the report. The train model accuracy is 0.996, 0.97, and 0.98 percent, respectively, while the train model loss is 0.01, 0.03, and 0.02 percent. In this scenario, the model loss is when you classify the result using an algorithm some data don’t detect that’s why the loss of some data.

#### Evaluation and performances

Figure 20 compares the accuracy of different algorithms with our algorithm result for a different year. This research uses different year email data outcomes and compares them with our execution result. Use different datasets and label data file sizes to detect and evaluate the algorithm's performance. Depending on the size of the dataset, the accuracy of every line in the diagram changes. According to this dataset d_s_, this paper's execution measurement and performance are better than other classification reports. These algorithms thoroughly examine the phishing email attack classification in this paper to generate a more accurate classification report and generate the result. The graph below shows that our classification results and reports are stronger than those of another algorithm after conducting experiments and comparing our execution time result with other mentioned reports. Figure 20 shows the comparative system report of our paper.

## Conclusion and future work 

This paper discusses a technique for classifying emails as phish and not phish and email phishing attacks with the help of machine and deep learning algorithms. The dataset was preprocessed and converted to a suitable design that could be used to create classifiers using features taken from the dataset. The chosen features are retrieved using (RE) regular expression and NLP language in Python programming. These are grouped in an appropriate organizer, divided into multiple classifiers. The SL and DL algorithms were employed, which require an arrangement set to sort the test set. To partition the dataset and cross-endorsement plan has been used. SVM, NB, and LSTM classifiers are used to detect a phishing attack. The system reports were used to get the best execution time results, with 0.996%. 0.97% and 98% accuracy rates. The email phishing attacks are explained in this work. You may quickly check for phish or non-phish data using the specified feature. After dividing phish or non-phish data, use text processing to eliminate the error. The phishing attack was detected and removed using supervised learning SVM, NB, and deep learning LSTM algorithms, with the greatest execution time and the classification report result. The suggested structure can be improved in the future by merging both phish and ham to establish a new unique dataset. The approach would be closer to the real-life scenario where fraudsters are regularly improving their techniques by changing up email formats, both phished and non-phished. We could pass on a consistent framework that could be used across associations and confidentially protect customers against phishing attacks if we used models.
